# Designed Minibinders Rewire Receptor Signaling to Enable Functional Human Myogenic Reprogramming

**DOI:** 10.64898/2026.04.26.720818

**Published:** 2026-04-27

**Authors:** Riya Keshri, Zachary Foreman, Phillip Barrett, Alexander Robinson, Gabriela Reyes, Ashish Phal, Aditya KrishnaKumar, Ethan Narog, Melodie Chiu, Shruti Jain, Xinru Wang, David Lee, Marc Exposit, Mohamad Adedi, Alec ST Smith, Sanjay Srivatsan, Jay Shendure, Julie Mathieu, David L Mack, David Baker, Hannele Ruohola-Baker

**Affiliations:** 1. Department of Biochemistry, University of Washington, Seattle, United States.; 2. Institute for Stem Cell and Regenerative Medicine, University of Washington,School of Medicine, Seattle, United States.; 3. Department of Biology, University of Washington, Seattle, United States.; 4. Department of Genomics, University of Washington, School of Medicine, Seattle, United States.; 5. Basic Sciences Division, Fred Hutchinson Cancer Center, Seattle, Washington, USA; 6. Institute for Protein Design, University of Washington, School of Medicine, Seattle, United States.; 7. Department of Comparative Medicine, University of Washington, Seattle, United States.; 8. Department of Neurobiology and Biophysics, University of Washington, Seattle, WA, USA; 9. Howard Hughes Medical Institute, University of Washington, Seattle, WA 98195, USA; 10. Department of Bioengineering, University of Washington, Seattle, WA, USA

## Abstract

Sarcopenia, loss of muscle mass is a considerable health burden that demands immediate societal attention. Direct myogenic somatic cell reprogramming, a potential muscle regeneration method is constrained by an inability to control the signaling logic that governs cell fate. Here, we show that this barrier can be overcome using AI-designed receptor modulators. Screening de novo minibinders, we identify a synthetic protein cocktail, C6-DPC, that drives efficient human fibroblast-to-muscle transdifferentiation with robust structural and metabolic maturation. C6-DPC reprograms extracellular signaling by activating pro-myogenic FGFR1/2c pathways while suppressing anti-myogenic inputs through ALK1 and TGFBR2; targeted depletion of ALK1 is sufficient to lower the reprogramming barrier. Inflammatory signaling via gp130 emerges as a dominant checkpoint, and its inhibition further enhances conversion. Engineered tissues generate high twitch and tetanic forces in both wild-type and dystrophin-deficient human cells. These findings demonstrate that programmable synthetic ligands can rewrite receptor-level signaling to direct cell fate and enable functional tissue regeneration.

## Introduction

Skeletal muscle strength and function begin to decline as early as the third decade of life, initiating a progressive trajectory toward sarcopenia, a major and growing health burden in the aging population (Pabla et al.,2024). Under physiological conditions, muscle homeostasis is sustained by satellite cells; however, their regenerative capacity diminishes sharply with age and can be further compromised by severe trauma, surgical injury, and genetic disorders such as Duchenne muscular dystrophy. Moreover, the clinical success resulting in population level use of GLP-1 receptor agonists (e.g., semaglutide, tirzepatide) can involve a significant reduction in lean body mass (LBM), which may exacerbate or trigger early sarcopenia (Scott et al.,2025; Langer et al., 2026).

Direct cellular reprogramming from one adult cell type to another has shown to occur in nature (Wu et al., 2012; Thorel et al., 2010). This process has recently been harnessed for regenerative medicine, offering a path to bypass the complexities of pluripotency while providing a source of patient specific functional cells. The conversion of fibroblasts into myogenic lineages via the ectopic expression of MyoD was the seminal demonstration of cell fate plasticity (Weintraub et al., 1987; Morris & Daley, 2013). However, despite decades of research, the therapeutic application of direct reprogramming is still hampered by low conversion efficiency (Cacchiarelli et al., 2015; Kim et al., 2022). Although MyoD established the principle of cell fate plasticity, its practical application remains limited by inefficient conversion and incomplete maturation. These limitations reflect a fundamental issue: cell fate is not governed by transcription factors alone, but by a complex extracellular signaling environment that needs to be controlled (Boularaoui et al., 2018; Cacchiarelli et al., 2018). Competing cues, including pro-fibrotic pathways actively oppose myogenic conversion, while current small-molecule approaches lack the specificity to resolve these conflicts. Furthermore, restricted chromatin accessibility limits MyoD binding to key regulatory elements and results in incomplete transcriptional activation of the myogenic program (Manandhar et al., 2017; Kolundzic et al., 2018; Liu et al., 2018). With small molecule approaches, dedifferentiation into induced myogenic progenitor cells (iMPCs) has also been explored, however these cells can remain highly proliferative (Yagi et al., 2021; Kim et al., 2022).

Recent breakthroughs in AI based molecular modeling, such as RFDiffusion and BindCraft, have introduced a new class of therapeutic agents: synthetic minibinders (Watson et al., 2023; Pacesa et al., 2025). These *de novo* designed proteins can be engineered with exquisite specificity down to the receptor isoform level and can function as either potent antagonists in monomeric form or “superagonists” when organized into high valency oligomeric scaffolds (Cao et al, 2022; Edman et al., 2024; Zhao et al., 2021). Unlike natural ligands, these minibinders can be displayed on rigid or flexible scaffolds to precisely tune signaling geometries. Recent work has already demonstrated their power; for example, FGFR1/2c-specific minibinders can switch the fate of pluripotent stem cells between endothelial and pericyte states (Edman et al. 2024).

In this study, we screened a library of AI-designed proteins to identify the novel protein cocktail that can overcome the efficiency bottleneck of myogenic conversion. We report that a strategic combination of minibinders specifically targeting the activation of FGFR1/2c alongside the inhibition of TGFBRII and ALK1R, significantly increases the efficiency and maturation of human fibroblast derived muscle cells. In parallel, inhibition of inflammatory signaling via gp130 removes a dominant barrier to conversion. Our results demonstrate that these synthetic protein cocktails not only refine the reprogramming process but also facilitate the functional rescue of DMD muscle force phenotypes, marking a new era in precision regenerative therapy.

## Results

### Designed receptor modulatory cocktail (DPC) enhances the skeletal muscle direct reprogramming

In this study we utilize designed proteins to target receptor tyrosine kinases or receptor serine/threonine kinases to make the human myogenic cell fate conversion efficient. We engineered neonatal skin fibroblasts (HFFs, human Foreskin Fibroblasts) to generate an inducible MyoD line (HFF-iMYOD) under the control of a Tet-on promoter (Fig-S1A). The continuous doxycycline-induced overexpression of MyoD in HFF-iMYOD for 7 and 14 days resulted in a low conversion of fibroblasts to myogenic lineage (Fig-S1C-G). Transcriptomic analysis revealed three distinct cell populations; resting fibroblasts maintaining baseline markers (MME, CXCL12), a population of activated myofibroblasts enriched for fibrotic markers (FN1, IGFBP5), and a reprogrammed cluster expressing muscle structural and functional genes (ACTC1, CHRNA1) (Fig-S1H).

We analyzed a set of barrier genes (ID1, ID2, ID3, TGFB1, TGFB2, TGFBR1, TWIST1, TWIST2, ZEB1, SNAI1, SNAI2, HES1, HEY1, NOTCH1, WNT5A) previously reported to inhibit myogenic differentiation through repression of MyoD activity, activation of Epithelial-Mesenchymal Transition (EMT) programs, or maintenance of progenitor states via TGF-β and Notch signaling pathways (Benezra et al., 1990; Spicer et al., 1996; Buas et al., 2009; Soleimani et al., 2012; von Maltzahn et al., 2012). Our analysis identified a hierarchical “brake” system categorized into four distinct regulatory drivers that actively antagonize myogenic transdifferentiation (Fig-S1I). The most potent barrier was the structural reinforcement of the extracellular matrix (ECM), characterized by strong positive correlations with *COL1A2*, *SPARC*, and *COL3A1* suggesting the physical rigidity and integrin signaling of the matrix structurally anchor cells to a non-myogenic identity. Activated myofibroblast markers, including *TAGLN*, *CTGF*, *INHBA*, and *POSTN*, showed significant positive correlation with the barrier score, confirming that a competing pro-fibrotic program directly diverts cells from the skeletal muscle fate (Fig-S1J). We identified active molecular inhibitors of myogenesis, specifically *IGFBP5*, *IGFBP7*, and *ID3* (Fig-S1J), and also identified a novel category of myogenic inhibitors, regulators of cellular stress and inflammation (*IFI6*, *STAT3*, and *NFKB1*). Collectively, these results map a multi-layered roadblock to myogenesis, where successful reprogramming requires the simultaneous suppression of structural ECM traps, competing fibrotic lineages, transcriptional repressors, and inflammatory stress states (Fig-S1J).

**Figure 1.**
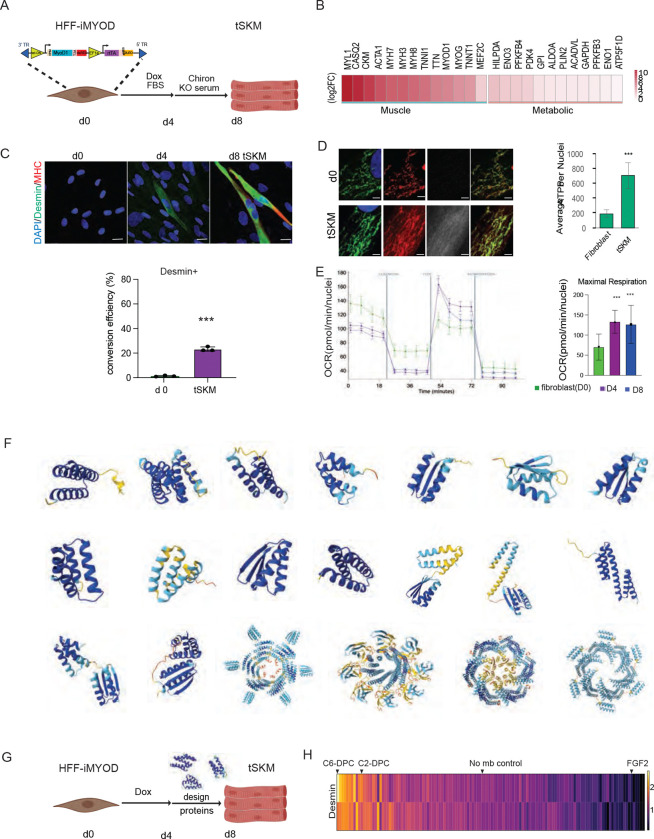
Direct reprogramming of human foreskin fibroblast into skeletal muscles. (A) Tet-on inducible MyoD Human Foreskin FIbroblast were treated with Doxycycline for 4 days, then treated with designed proteins & chiron in KO serum media for the following 4 days to reprogram into muscle cells, where immunofluorescent staining for muscle markers was performed. (B) a heatmap showing 8 day tSKM versus fibroblast bulk sequencing data where muscle markers and metabolic gene expression are increased. (C) D8 (D4>8) reprogrammed tSKM shows muscle markers staining for Desmin (green), Myosin heavy chain (red), DAPI (blue). A bar graph of conversion efficiency (desmin+) of D8 tSKM vs uninduced HFFs. scale bar=30um (D) Confocal images of uninduced fibroblast and day 8 tSKM showing mitochondrial marker ATP-b synthase(green), mito-Orange (red) uptake, Desmin (grey) and DAPI(blue). A graph showing ATP-b synthase intensity in uninduced fibroblast, day8 no mb tSKM, day8 DPC treated tSKM. scale bar=5um (E) Normalised OCR measure in uninduced fibroblast, day4 and day8tSKM. Maximum respiration measured in uninduced fibroblast, day4 and day8tSKM. (F) Few examples Alphafold3 predicted stuctutre of design binders used in the muscle transdifferenation. (G) schematic showing minibinder screen in skeletal muscle transdifferentiation. (H) A heatmap showing two independent screens of minibinders in 8day tSKM. Where desmin intensity in twoindependent primary screens of designed proteins at 100nM concentration in iMYODHFF has been plotted.

To date, numerous biological ligands including Follistatin, GDF8, FGF2, GDF11, GDF15, hGH, TMSB4X, BMP4, BMP7, IL-6, and TNF-a have been evaluated for their ability to enhance MyoD induced transdifferentiation process, but none have proven successful (Abdel-Raouf et al., 2021). However, these factors predominantly promote cellular proliferation or function as negative regulators that further impede myogenic conversion. To identify novel potent signaling pathways beneficial for direct myoblast reprogramming, we established a streamlined, defined serum-free screening assay and screened for novel AI-designed proteins that enhanced the direct reprogramming. We conducted a temporal screen to define the minimal Dox exposure required to prime the fibroblasts for myogenic reprogramming. Following induction, cells were transitioned into serum-free media for various periods to observe the progression of differentiation (Fig-1-S1K). Our time-course analysis revealed that a 4-day Dox pulse (D4) followed by a 4-day release into serum-free media (D8) was sufficient to drive consistent, albeit still low myogenic conversion (Fig-1A).

Under these stable D8 conditions, approximately 20% of the population expressed the intermediate filament Desmin and Myosin heavy chain (Fig-1B-C, Fig-1-S1L). Additionally, we observed significant metabolic remodeling, a hallmark of the transition from a fibroblastic state to a mature myogenic oxidative profile (Fig-1D-E). The Desmin⁺ cells showed elevated ATPB expression (Fig. 1D) and both D4 and D8 stages exhibited significantly enhanced mitochondrial maximal respiration compared to the starting fibroblast population (Fig. 1E). Thus, the D8 state captures both myogenic markers and the essential metabolic switch for functional muscle. However, conversion efficiency remained low (~20%, Fig. 1C), making this an ideal platform to screen AI-designed minibinders with improved myogenic capacity.

Leveraging recent advances in computational protein designs (Fig. 1F), we hypothesized that precise outside-in signaling control using engineered minibinders could overcome fate stabilizing barriers and promote productive myogenic reprogramming (Keshri et al., 2024). We therefore employed an optimized D8 reprogramming paradigm, in which minibinders were applied under serum-free conditions between day 4 and day 8, a critical window for fate stabilization (Fig-1G-H). We first performed a systematic screen of individual minibinders targeting FGFR, ALK1R, PDGFR, InsulinR, IGF1R, TGFBRII, and EGFR as monomeric antagonists, together with engineered agonists including FGFR-C6, TRKA-C2, Tie2-H8, and EGFR (Table 1) (Cao et al. 2021; Edman et al.,2024; Schlichthaerle et al., 2025; Zhao et al., 2021). We analyse the desmin intensity of each condition as a readout for myogenic reprogramming (Fig-1H, Table 2). From this screen, we identified two key design protein cocktails (DPC) that significantly enhanced myogenic conversion. : (I) C6-DPC (TGFBRIImb antagonist + ALK1Rmb antagonist + FGFR-C6 agonist) and (II) C2-DPC (TGFBRIImb antagonist + ALK1Rmb antagonist + TRKA-C2 agonist) where ALK1 and TGFBR2 pathway inhibition remains central in both the DPCs (Fig-3A).

### Alk1 pathway strong suppression is sufficient for efficient myogenic transdifferentiation

**Figure 2.**
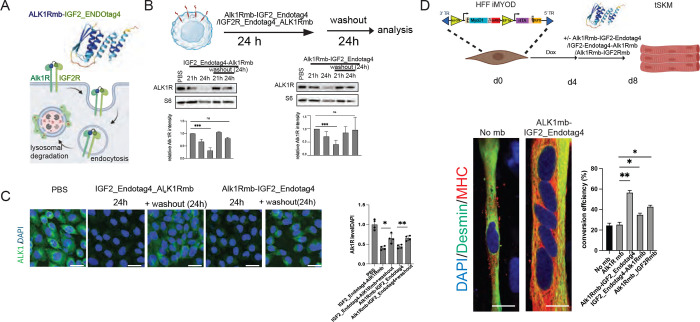
Strong degradation of ALK1R augments transdifferentiation. (A) Alk1Rmb-IGF2R_Endotag4 fusion directs the Alk1 receptor together with IGF2R endosomal internalization, degraded through lysosomal trafficking. (B) CHOcells overexpressing the human Alk1R are treated with the Alk1Rmb-IGF2R_Endotag4mb or IGF2R_Endotag4mb-Alk1Rmb for 24hrs. and washed out for another 24 hrs., cells are analysed for Alk1R expression by western blot analysis. The bar graph shows significant recovery of Alk1R after recovery. (C) Immunofluorescence imaging shows Alk1R (green), phalloidin (red) and DAPI (blue) staining in the CHO cells expressing the Alk1R-intensity. Scale bar=50um (D) Immunofluorescence imaging shows reprogrammed tSKM under no mb (control) and Alk1-IGF2R_Endotag4 treatment shown in the schematic stained for muscle markers staining for Desmin (green), Myosin heavy chain (red), DAPI (blue) in PBS and Alk1. A bar graph showing fibroblast to muscle conversion efficiency is significantly upregulated in Alk1-IGF2R_Endotag4 treatment. Scale bar=15um

To validate the mechanism of the designed receptor modulatory cocktails, we focused on the inhibition of the Alk1 pathway. We utilized the IGF2_EndoTag4-Alk1 Novokine to test Alk1 inhibition by an alternative modality (Li et al., 2025). IGF2_EndoTag4 minibinder facilitates the endosomal internalization and subsequent lysosomal degradation of bound ligand complexes (Fig-2A). We therefore engineered an IGF2_EndoTag4-Alk1mb fusion designed to deplete ALK1 protein from the plasma membrane. We tested two orientations of the fusion proteins Alk1mb: IGF2_EndoTag4 mb (A1IE) and IGF2_EndoTag4 mb: Alk1mb (IEA1). To assess their efficacy, we treated CHO-human ALK1 cells for 24 hours and observed that both A1IE and IEA1 fusions induced a significant reduction in hALK1R protein levels (Fig-2B-C). ALK1R levels returned close to baseline 24 hours after the removal of the fusions, confirming the specificity and reversibility of the EndoTag4 system (Fig-2B-C).

We tested the A1IE in the transdifferentiation assay (Fig-2D). Importantly, A1IE treatment significantly increased both myogenic transdifferentiation efficiency and myotube length, further supporting the conclusion that repression of Alk1 signaling pathway enhances myogenic transdifferentiation (Fig-2D),

The higher activity of Alk1mb–IGFR2-EndoTag4 compared with Alk1mb alone indicates that physical depletion of ALK1 from the cell surface lowers the reprogramming barrier more effectively than antagonizing its activity by a minibinder. More broadly, our results establish AI-designed minibinder-driven targeted protein degradation as a powerful strategy to remove inhibitory signaling nodes. Using this approach, we identify ALK1R and TGFBRII as major molecular roadblocks to efficient muscle transdifferentiation and demonstrate that their programmable elimination can unlock latent myogenic potential.

### C6-DPC and C2-DPC accelerate skeletal muscle maturation

**Figure. 3.**
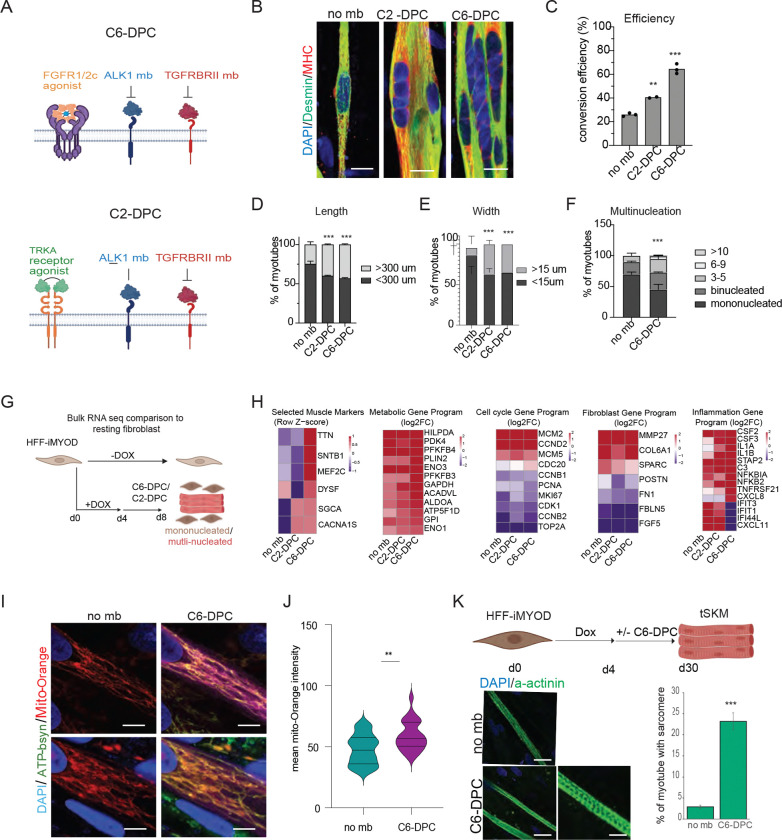
Designed protein cocktail enhanced myogenic transdifferentiation. (A) A schematic showing the two key designed protein cocktails, C6-DPC and C2-DPC. (B) Immunofluorescence images showing No mb, C2-DPC, and C6-DPC treated 8 days old tSKM stained for Desmin (green), MHC (red) and DAPI (blue). Scale bar=15um (C) Bar graph showing significantly increased conversion efficiency in C2-DPC and C6-DPC treated fibroblasts. (D) Length of myotubes significantly increased in C2-DPC and C6-DPC treated tSKM. (E) Width of myotubes significantly increased in C2-DPC and C6-DPC treated tSKM. (F) Multinucleation of myotubes significantly increased in C6-DPC treated tSKM. (G) Schematic showing Bulk RNA sequencing of tSKM and the mononucleated unconverted cells. (H) Heatmap showing muscle, metabolic, Cell cycle gene, fibroblast and inflammation markers expression of transcript level in no mb (control), C2-DPC, C6-DPC treatments versus fibroblast during the D8 conversion. (I) Confocal images of uninduced fibroblast, day8 no mb tSKM, day8 C6-DPC treated tSKM showing mitochondrial marker ATP-B synthase (green), mito-Orange (red) uptake, Desmin (magenta) and DAPI (blue). scale bar=5um (J) Violin plot showing mito-Orange intensity in day8 no mb tSKM, day8 C6-DPC treated tSKM. (K) Schematic of 30 day myotube maturation experiment. Sarcomere formation analysed with alpha-actinin staining(green) in day30 no mb tSKM and DPC treated tSKM. scale bar=15um and 5um

We analyzed the two powerful minibinder cocktails, C2-DPC and C6-DPC (Fig-3A). When compared to non-minibinder treated controls, myotubes treated with the C6-DPC or C2-DPC exhibited several key hallmarks of advanced maturation (Fig-3B). A higher percentage of fibroblasts successfully transitioned into the myogenic lineage in C6-DPC treatment, surpassing 3x over the ~20% baseline observed in standard conditions (Fig-3C). There was a significant increase in both the length and width of the resulting myotubes, indicating more robust protein synthesis and sarcomeric assembly (Fig-3D-E). Treated cells showed a significant increase in fusion index by both the DPCs, characterized by the presence of multiple nuclei within a single continuous cytoplasm, a critical step for functional muscle contraction (Fig-3F). While both cocktails outperformed the control, the C6-DPC (FGFR1/2c agonist + ALK1R and TGFBRII antagonists) consistently produced the most mature phenotypes.

### C6-DPC enhances myogenic remodeling

Transcriptomic analysis (Fig 3G) revealed that both C2-DPC and C6-DPC induce MyoD-mediated fibroblast reprogramming leading to muscle marker expression (MYH7, ACTA1, TTN, MYL4, CASQ2 etc) and reduced expression of fibroblast markers (FBLN5, FN1, FGF5) (Fig-S3A; Fig. 3H). Both C2-DPC and C6-DPC conditions further induced a myogenic reprogramming response, as demonstrated by the upregulation of skeletal muscle associated genes (LMOD3, MYL6B, COX6A2, PDLIM4) involved in sarcomere assembly and contractile function (Fig-S3B). Furthermore, metabolic genes were upregulated, and cell cycle genes downregulated, indications of C2-DPC and C6-DPC capacity to accelerate maturation in direct reprogramming (Fig. 3H).

In addition to C6-DPC inducing sarcomeric and cytoskeletal assembly genes such as LMOD3, and SNTB1, together with mitochondrial and oxidative metabolism components including COX6A2 and ACADVL (Fig-3H), we identified a new category of affected genes: C6-DPC specifically suppresses the interferon/inflammatory barrier (IFIT1, IFIT3, IFI44L, CXCL11) (Fig-3H). Some inflammatory and NF-κB-associated genes (such as IL1A, IL1B, IL24, IL32, CSF2, CSF3, CXCL8, NFKBIA, NFKB2, STAP2, and TNFRSF21) remained significantly upregulated under C6 conditions, indicating that myogenic reprogramming occurs alongside a persistent inflammatory transcriptional program (Fig-3H). C6-DPC does not globally suppress stress-associated programs, instead, biased FGFR signaling combined with ALK1R and TGFBR2 inhibition selectively enables myogenic structural and metabolic maturation to dominate over fate-stabilizing fibroblast programs. These findings support a model in which efficient lineage conversion is achieved not by erasing inflammatory identity, but by functionally overriding it through targeted signaling bias that unlocks irreversible myogenic commitment. To confirm the metabolic remodeling, we show that C6-DPC induced increased mitochondrial Mito-orange uptake indicating improved bioenergetic capacity (Fig-3I-J). Further, prolonged C6-DPC treated cells exhibited pronounced sarcomere organization and myofiber maturation (Fig-3K). These data indicated that C6-DPC FGFR1c-biased signaling as a dominant determinant of functional myogenic reprogramming.

### Inflammation perturbs promyogenic signal

**Figure 4-:**
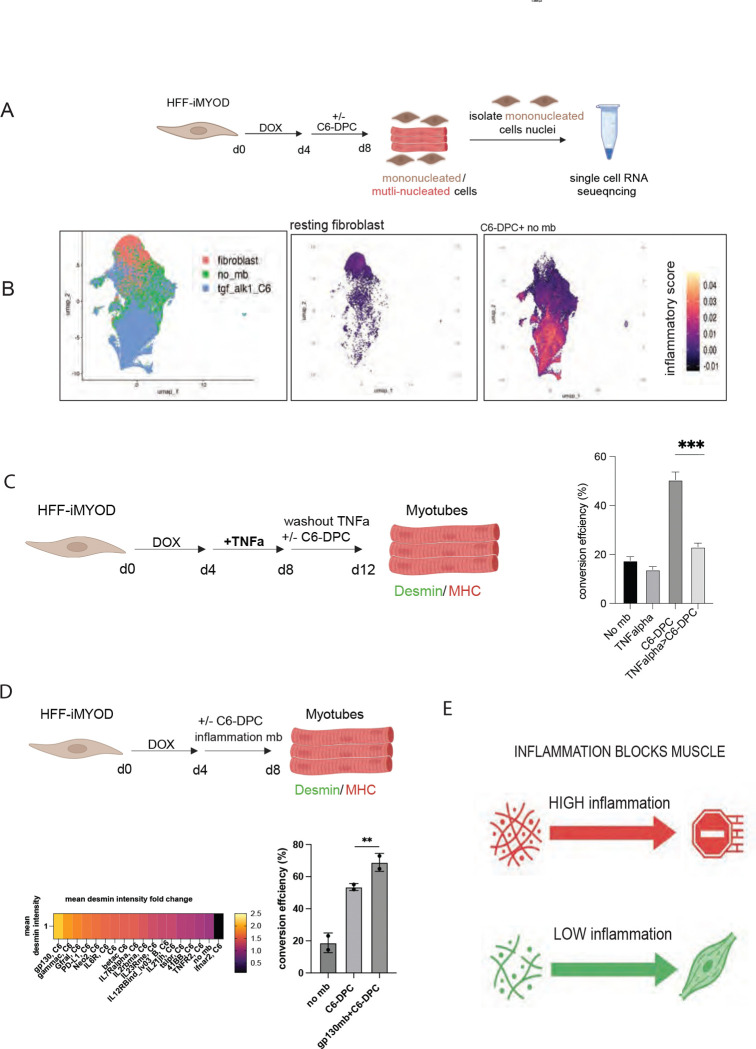
Inflammation blocks muscle reprogramming. (A) Schematic showing single cell RNA sequencing of original resting fibroblast, D8 tSKM no mb and D8 tSKM C6-DPC treated mononucleated cells. (B) UMAP showing all the nuclei sequenced. UMAP showing the fibroblast (left) and, no mb and C6-DPC (combined, right) inflammation score based on inflammatory cytokines and receptor expression level. (C) HFF-iMYOD were induced with doxycycline for 4 days, followed by TNF-alpha treatment for next 4 days to induce inflammation followed by treatment of C6-DPC for 4 days. Bar graph showing fibroblast to muscle conversion efficiency in various treatments from immunofluorescence of desmin and DAPI. (D) HFF-iMYOD were induced with doxycycline for 4 days, followed by treatment of C6-DPC and inflammatory minibinders for another 4 days. Heatmap showing mean desmin intensity fold change of two independent minibinder screens. Bar graph shows fibroblast to muscle conversion efficiency in C6-DPC is enhanced with gp130 inhibition. (E) A schematic model showing the conversion of fibroblast to skeletal muscles requires low inflammation.

Single-cell transcriptomic profiling of cells that did not commit to a myogenic fate revealed marked induction of inflammatory pathways (Fig-4A, 4B), such as TNFRSF19 and TNFRSF21. Elevated TDO2, PLAT, and MMP expression in non-committed C6-DPC treated cells further indicates activation of a TNF-associated, stress-adapted, ECM-remodeling transcriptional program (Fig-S3D). To investigate the impact of the inflammatory “roadblock”, we challenged the C6-DPC with TNF-a treatment (Fig-4C; Zhao et al., 2015). While the C6-DPC typically drives high-efficiency myogenic conversion, the addition of TNF-a fully prevented the C6-DPC dependent increase in conversion efficiency (Fig-4C). To overcome this high inflammation and fibrosis roadblock for myogenic transdifferentiation, we screened for inflammation inhibitors using a library of designed minibinder antagonists against IL- and interferon-receptor pathways (Table 3). While further interferon pathway inhibition did not increase C6-DPC conversion efficiency, inhibiting the gp130 receptor with a designed minibinder (gp130mb) significantly enhanced myogenic conversion efficiency of C6-DPC (Fig-4D, (Table 4). This finding suggests that inflammatory stress acts as a dominant inhibitory checkpoint that can override the pro-myogenic signals provided by FGFR1/2c activation and ALK1R/TGFBRII inhibition (Fig-4E). These findings are supported contextually with previous studies (Berkes and Tapscott, 2005; Cao et al., 2010; Guttridge et al., 2000; Langen et al., 2004; Cai et al., 2004) showing that TNF-α signaling interferes with MyoD transcriptional activity and disrupts activation of the myogenic feed-forward network, while gp130-IL6–STAT3 signaling reinforces a competing fibro-inflammatory transcriptional state that overrides differentiation enforcing a non-myogenic cell state (Guttridge et al., 2000; Serrano et al., 2008; Tierney et al., 2014).

### Designed protein cocktail promotes iPSCs into muscle differentiation

**Figure 5.**
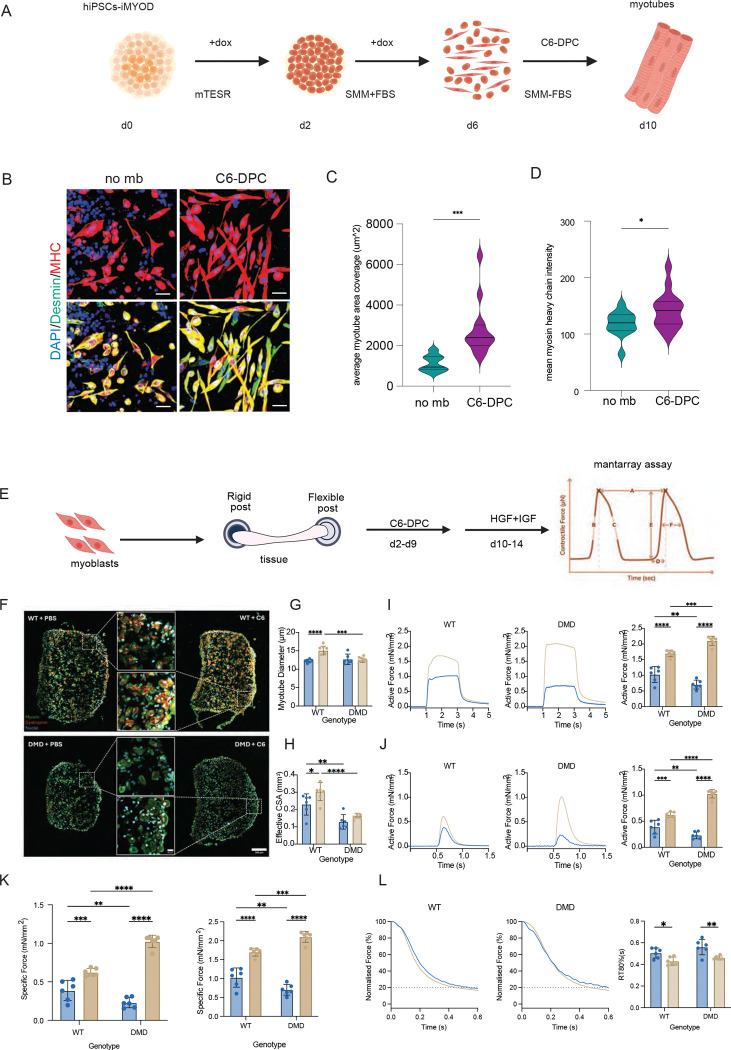
C6-DPC treatment enhances iPSCs differentiation into skeletal muscles and enhances contractile force and accelerates kinetics in iPSC-derived EMTs. (A) Schematic showing iPSCs-iMYOD differentiation into myotubes in C6-DPC for 4 days and fixed and stained for muscle makers. (B) iPSCs differentiated into myotubes are stained for DAPI (blue), desmin(green) and MHC (red). Scale bar= 10um. (C) Violin plot showing average myotube area coverage enhanced in DPC treatment versus no minbinder treatment. (D) Mean intensity of MHC shows increase in DPC treatment versus no minbinder treatment. (E) Schematic showing mantarray assay measuring the contractile forces where the 3D muscle tissue (EMTs) is scaffolded around a rigid and a flexible post. The contractile force generated by the tissue (is measured the displacement of a magnet in the flexible post. **(F)** Representative immunofluorescence cross-sections of WT and DMD engineered muscle tissue (EMTs) treated with PBS (vehicle) or C6-DPC (100 nM). Sections are stained for myosin heavy chain (green), dystrophin (red), and nuclei (blue). Scale bar: 200 μm. Insets show higher magnification of myotube architecture (Scale bar: 20 μm). **(G)** Representative tetanic (100Hz) force traces (left) and quantification of specific tetanic force (right), normalized to effective CSA. **(H)** Representative twitch (0.2Hz) force traces (left) and quantification of specific twitch force (right), normalized to effective CSA. **(I)** Quantification of Effective Cross-Sectional Area (CSA), representing the total area occupied by myosin-positive myotubes (left), and quantification of average myotube diameter (right). C6-DPC induces hypertrophy in WT but not DMD myotubes. **(J)** Normalized relaxation traces (left) and quantification of the time to 80% relaxation (RT80%) (right), indicating improved relaxation kinetics with C6-DPC treatment. Data are presented as mean ± S.E.M. *P < 0.05, **P < 0.01, ***P < 0.001, ****P < 0.0001 by two-way ANOVA with Tukey’s multiple comparisons test.

To determine if the C6-DPC has a general myogenic capacity, beyond transdifferentiation, we analyzed the cocktail in iPSC derived skeletal muscle differentiation protocol. WTC11(doxMyoD) human iPSCs were treated with doxycycline to initiate myogenic commitment, followed by differentiation in skeletal muscle medium and subsequently treated with C6-DPC (Fig-5A). Importantly, C6-DPC treatment resulted in robust formation of multinucleated myotubes expressing canonical muscle markers desmin and Myosin Heavy Chain (MHC). Quantitative analysis revealed that C6-DPC treatment significantly increased myotube area coverage relative to control conditions lacking minibinders and the mean fluorescence intensity of MHC was significantly elevated in C6-DPC treated cultures (Fig-5C-D). These data indicate that C6-DPC increases sarcomeric protein accumulation and myofiber maturation during iPSC derived muscle differentiation.

### C6-DPC enhances structural and functional maturation of iPSC-derived engineered muscle tissues

To determine whether C6-DPC induces functional gains in a 3D muscle tissue context, we generated engineered muscle tissues (EMTs) from both wild-type and dystrophin-deficient (DMD) iPSC derived myoblasts in the presence or absence of C6-DPC and assessed contractile function using the Mantarray magnetometric platform (Fig. 5E). Immunofluorescence cross-sections of C6-DPC-treated EMTs revealed denser, more organized myofiber architecture compared to PBS-treated controls in both genotypes (Fig. 5F). We quantified this structural maturation, observing a significant increase in effective cross-sectional area (the total area with myosin positive myotubes; CSA) with C6-DPC treatment in wild type (PBS: 0.229 vs C6-DPC: 0.304 mm^2^) and in DMD EMTs (PBS: 0.128 vs C6-DPC: 0.165 mm^2^) (Fig. 5I). C6-DPC treatment also increased mean myotube diameter in WT tissues (PBS: 12.24 vs C6-DPC: 15.06 μm), though this hypertrophic effect was not significant in DMD EMTs (Fig. 5I), suggesting that the increased CSA in DMD tissues reflects enhanced myoblast fusion and fiber density rather than individual fiber hypertrophy.

Critically, these structural gains translated directly into enhanced intrinsic contractile output. To accurately assess muscle quality independent of the larger tissue cross-section, we evaluated specific force by normalizing the active force to the effective CSA. Under tetanic stimulation (100 Hz), C6-DPC treated EMTs exhibited significantly elevated specific force, generating approximately 1.6-fold greater than control force in WT tissues (PBS: 1.03 vs C6-DPC: 1.69 mN/mm^2^) and a profound 3 fold increase in DMD tissues (PBS: 0.70 vs C6: 2.10 mN/mm^2^) (Fig. 5G). Single twitch responses (0.2 Hz) followed a similar pattern, C6-DPC treatment increased specific force by ~1.6 fold in WT and ~4.5 fold in DMD EMTs specific twitch force (Fig. 5H). This demonstrates that the contractile improvement exceeds what can be attributed to increased tissue size alone, indicating that C6-DPC treatment fundamentally enhances the force-generating capacity of the myofibers themselves.

To further assess functional maturation, we examined relaxation kinetics following tetanic contraction. C6-DPC treatment significantly accelerated the time to 80% force relaxation (RT80%) in both WT (PBS: 0.505s vs C6-DPC: 0.430s, Δ75 ms) and DMD tissues (PBS: 0.560s vs C6-DPC: 0.460s, Δ100 ms) (Fig. 5J). Faster relaxation is a hallmark of mature skeletal muscle, reflecting efficient calcium reuptake and cross-bridge detachment kinetics.

Collectively, these results demonstrate that C6-DPC does not simply produce larger muscle; it produces muscle with intrinsically greater force. The convergence of increased specific force, accelerated relaxation kinetics, and enhanced myofiber fusion defines a coordinated maturation program that is preserved across both healthy and dystrophin-deficient backgrounds, establishing synthetic receptor-specific signaling as a viable strategy for functional muscle regeneration.

### Cooperative regulation of promoter accessibility enhanced during C6 cocktail induced myogenic reprogramming

**Figure-6.**
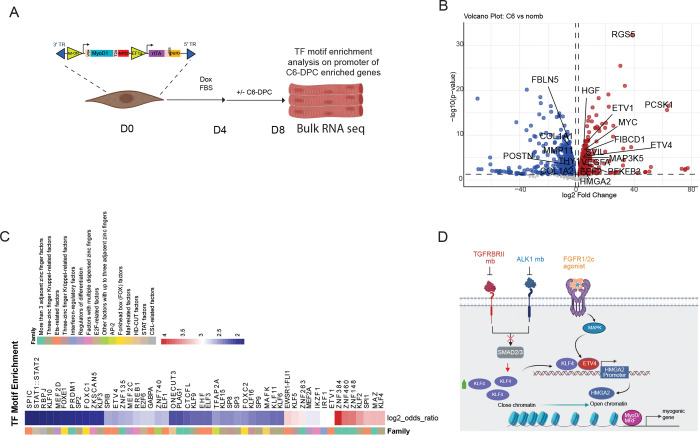
C6-DPC mediates promyogenic chromatin remodeling. (A) A schematic showing C6-DPC and no mb treated tSKM sent for bulk sequencing. (B) A volcano map showing genes upregulated (red) or downregulated (blue) in D8 tSKM in C6-DPC treated tSKM versus no mb tSKM. (C) A heatmap showing upregulation of top 50 transcription factors motifs enriched in the promoter of 185 upregulated genes. (D) Proposed model showing C6-DPC mediated enhancement of myogenic reprogramming. Inhibition of TGFBR2/ALK1 suppresses SMAD2/3 signaling and stabilizes KLF4, while FGFR1/2c agonism induces MAPK-ETV4 signaling. Cooperative KLF4-ETV4 activity promotes HMGA2-driven chromatin opening, enabling activation of myogenic gene programs.

Bulk RNA sequencing revealed that treatment with the designed protein cocktail (C6-DPC) induces a robust transcriptional program consistent with myogenic differentiation and muscle maturation in transdifferentiated skeletal muscle (tSKM) cells (Fig-6A-B). The C6-DPC treatment significantly upregulated genes associated with muscle progenitor activation, cytoskeletal remodeling, and paracrine signaling, including MSX1, HGF, SVIL, FIBCD1, SNAI1, CMKLR1, PTGS2, and PGF, reflecting a coordinated shift toward a pro-myogenic and growth-permissive state (Fig-6A-B). Gene Ontology enrichment and KEGG analysis revealed activation of PI3K-AKT, MAPK/ERK, JAK-STAT, calcium, VEGF, Wnt signaling, and arachidonic acid metabolism (Fig-S4A,B), while Reactome analysis highlighted enrichment of ERK/MAPK cascades, Notch signaling, and prostaglandin biosynthesis(Fig-S4C), collectively indicating enhanced growth factor responsiveness, survival signaling, and metabolic remodeling downstream of 6C-DPC treatment. Notably, induction of PTGS2 and downstream prostaglandin pathways, including prostaglandin F2 (Fig-S4E), aligns with established roles of prostaglandin signaling in muscle growth and regeneration (Ho et al., 2017; Wang et al., 2025). The upregulation of HGF and the myokine FIBCD1 (Fig-6B) further suggests activation of autocrine and paracrine programs that support myofiber growth and tissue remodeling (Tatsumi et al., 1998; Graça et al., 2022), consistent with the increased myotube dimensions observed following 6C-DPC treatment. Developmental regulators such as ID3 and SNAI1 (Fig-6B), that are associated with early lineage plasticity and morphogenetic programs, were also elevated (Benezra et al., 1990; Soleimani et al., 2012), suggesting partial reactivation of developmental transcriptional states that may synergize with chromatin remodeling to enhance maturation (Fig-S4D).

To identify transcriptional regulators underlying enhanced myogenic reprogramming induced by the FGFR1/2c-agonist based C6 designed protein cocktail (C6-DPC), we performed motif enrichment analysis on regulatory regions associated with genes upregulated during conversion. We extracted promoter sequences for the 185 upregulated genes by defining each promoter as a fixed window spanning −2000 bp upstream to +200 bp downstream of the transcription start site (TSS). First, the 185 genes were mapped to their corresponding genomic coordinates and strand information using a reference genome annotation (e.g., hg38/GRCh38). Our motif enrichment analysis on regulatory regions associated with it revealed a significant enrichment of KLF family motifs, characterized by GC-rich CACCC elements, together with ETS family motifs, including the canonical GGAA sequence recognized by ETV1 and ETV4 (Fig-6C). Notably, KLF and ETS motifs frequently co-occurred within promoter-proximal and enhancer regions of genes implicated in cytoskeletal organization, metabolic remodeling, and early myogenic commitment. We propose a model in which coordinated inhibition of TGFβR and ALK1R signaling leads to reduced SMAD2/3 activity, resulting in stabilization and functional activation of KLF4 (Chen et al, 2026) which primes chromatin accessibility and can bind the HMGA2 promoter (Fig-6D). Concurrent FGFR1/2c mediated MAPK activation (Edman et al., 2024) induces ETS transcription factors, ETV1/4, which is shown to co-target HMGA2 regulatory regions. Upregulation of HMGA2 could drive global chromatin decompaction, removing epigenetic barriers and enabling efficient MyoD / other MRFs-driven activation of the myogenic program (Fig-6D).

We propose a model in which AI-designed receptor-modulatory minibinders and inflammation inhibitor minibinders rewire extracellular signaling to overcome barriers to myogenic reprogramming. In this framework, an engineered FGFR1/2c-specific agonist activates pro-myogenic MAPK/ERK signaling, leading to induction of ETS transcription factors that promote muscle gene expression and metabolic maturation. In parallel, inhibitory minibinders targeting TGFβ signaling components, including ALK1 and TGFBR2, suppress SMAD2/3-driven fibrotic and anti-myogenic programs that otherwise divert cells toward non-muscle lineages. Furthermore, inflammation inhibition by designed minibinders is beneficial for muscle maturation since TNF-α signaling disrupts activation of the MyoD-myogenic feed-forward network, and gp130-IL–STAT3 signaling reinforces a competing fibro-inflammatory transcriptional state that overrides differentiation enforcing a non-myogenic cell state (Guttridge et al., 2000; Serrano et al., 2008; Tierney et al., 2014).

**Figure 7.**
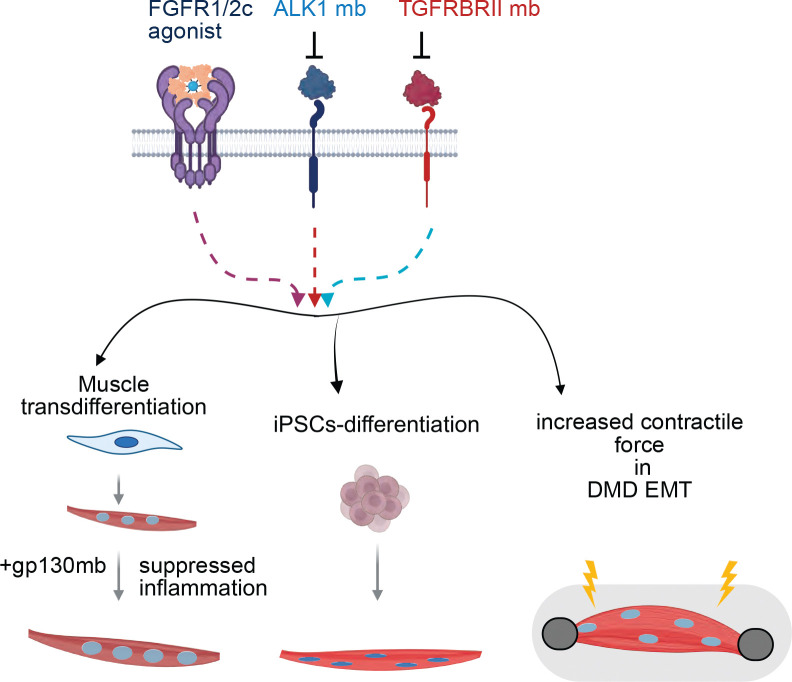
Promyogenic designed protein cocktail. A schematic showing enhanced muscle transdifferentiation, iPSC derived muscle differentiation and increased contractile forces in the 3D EMTs formed from the DMD-IPSC derived myoblast in presence of the C6-DPC.

The combined action of these signals creates a permissive transcriptional and chromatin landscape, enabling efficient MyoD driven conversion of fibroblasts into multinucleated myotubes. This rewiring of the signaling network does not only enhance structural differentiation, evidenced by increased fusion and sarcomeric organization but also promotes functional maturation, including elevated mitochondrial activity and improved contractile force in engineered muscle tissues (Fig-7). Notably, in dystrophin-deficient contexts, this approach enhances muscle performance despite the underlying genetic defect, highlighting the therapeutic potential of designed extracellular signaling to drive regenerative outcomes (Fig-7).

## Discussion

Our study demonstrates that programmable extracellular signaling using AI-designed synthetic minibinders can overcome key barriers that restrict myogenic transdifferentiation. By defining an optimized reprogramming window and applying single-cell transcriptomic analysis, we identified that the majority of fibroblasts resist conversion through a coordinated barrier network involving extracellular matrix reinforcement, fibrotic lineage commitment, and transcriptional inhibitors of myogenesis. This barrier landscape reveals that inefficient reprogramming is not a stochastic process but rather reflects a structured regulatory program that stabilizes fibroblast identity. Targeted manipulation of this barrier network using AI-designed receptor modulators proved sufficient to unlock productive lineage conversion. The superior performance of the C6-DPC combining FGFR1/2c agonism with inhibition of ALK1 and TGFBR2 signaling, demonstrates that efficient myogenic reprogramming requires simultaneous activation of pro-myogenic pathways and suppression of fate-stabilizing signaling. Unlike natural growth factors, which often trigger pleiotropic or proliferative responses, synthetic C6-DPC enables receptor isoform specific control of signaling architecture. This precision allows selective attenuation of fibrotic signaling programs while reinforcing metabolic and structural pathways associated with muscle differentiation.

This work resolves a longstanding question in the field by defining the critical, targetable nodes of small-molecule–mediated direct reprogramming. Prior studies identified SB431542 and LDN-193189 as effective modulators of TGFβ-family signaling; however, their lack of specificity and the breadth of the TGFβ superfamily have limited mechanistic clarity (Jeong et al.,2021). Here, we demonstrate that two AI-designed, pathway-specific inhibitors-Alk1mb and TGFβRmb-are sufficient to precisely suppress the key TGFβ-family components that constrain myogenic reprogramming. These findings move the field from correlative small-molecule effects to a defined, target-centric framework, establishing that selective inhibition of discrete signaling nodes can recapitulate-and refine-the pro-reprogramming activity of broadly acting compounds.

Our findings identify inflammatory signaling as a novel, dominant regulatory checkpoint in myogenic reprogramming. The potent inhibitory effect of TNF-α on C6-DPC–mediated conversion demonstrates that inflammatory pathways can override pro-myogenic cues, providing a mechanistic basis for impaired regenerative capacity observed in aging and muscular dystrophy. While RTK/RS–TK modulation drives unprecedented levels of transdifferentiation, conversion efficiency ultimately plateaus, revealing an intrinsic barrier. Strikingly, targeted suppression of inflammatory signaling lifts this ceiling: co-treatment with a designed gp130 antagonist significantly enhances myogenic conversion beyond this limit. Mechanistically, gp130 inhibition attenuates IL–JAK–STAT3 signaling (Selander et al., 2004; Sato et al.,2024), thereby dismantling pro-inflammatory and pro-fibrotic transcriptional programs that stabilize fibroblast identity and antagonize MyoD-driven lineage conversion. Together, these findings position inflammatory signaling as a central, targetable constraint on reprogramming and establish its modulation as a powerful complementary strategy to improve regenerative outcomes, particularly in diseased or aged tissues.

Importantly, the effects of C6-DPC extend beyond structural differentiation to intrinsic functional maturation. In engineered muscle tissues, C6-DPC treatment increased not only absolute contractile force but also specific force. Engineered tissues frequently produce increased force output that reflects greater cell mass rather than improved myofiber quality (Rao et al., 2018). The specific force analysis presented here directly addresses this distinction, demonstrating that C6-DPC enhances intrinsic force-generating capacity per unit cross-sectional area, providing evidence that receptor-specific signaling modulation drives contractile improvement at the myofiber level, not simply tissue expansion. This is accompanied by accelerated relaxation kinetics, a functional signature of mature calcium handling and cross-bridge cycling (Racca et al., 2013). C6-DPC-treated cells exhibited enhanced myotube maturation and significantly improved twitch and tetanic force generation, including those derived from dystrophin-deficient iPSCs. This functional rescue suggests that precise control of receptor signaling can partially compensate for the contractile deficits associated with several muscle-related disorders. While the primary focus of this work is the signaling framework rather than DMD therapeutics, the functional rescue raises the possibility that maturation-targeted interventions could complement dystrophin-restoration strategies where functional recovery often remains incomplete despite partial genetic correction (Hoffman et al., 2011; Oskoui et al., 2025).

Together, these findings establish programmable AI-designed receptor binders as a versatile platform for rewiring receptor signaling networks to control cell fate transitions. By combining RTK and IL pathway activation, inhibition, and targeted receptor degradation within a single design framework, AI-designed minibinders enable rational engineering of cellular signaling environments. This strategy provides a generalizable approach for overcoming lineage barriers and enhancing functional tissue regeneration, with potential applications in muscle repair, degenerative disease modeling, and precision regenerative medicine and mitigation of GLP-1 receptor agonist–associated lean mass loss. In this context, designed protein combinations could be tailored to preserve or regenerate skeletal muscle and reduce the risk of drug-associated sarcopenia.

## Materials and Methods-

### Cell culture- CHO cells, immortalized HUVEC cells, HFF-MyoD, Primary human myoblasts IPSCs

HFF-MyoD cells- HFF-MyoD cells were maintained in growth media (DMEM with 4.5g/L glucose and Glutamax [Gibco #10566024], 10% fetal bovine serum (FBS) [BioWest #S1620], 1% penicillin/streptomycin (P/S) [Gibco #15140122], 1% NEAA [Gibco #11140050]) at 37°C and 5% CO2. At 80% confluency HFF-MyoD cells were passaged and seeded at a 1:6 ratio.

### Expression and protein purification

Sequences of the designed proteins were reverse translated with optimization for Escherichia coli expression, with a C-terminal glycine-serine linker followed by a 6× histidine tag. Sequences were ordered as synthetic genes from Integrated DNA Technologies within the pET29b+ vector between NdeI and XhoI cloning sites. This vector contains a kanamycin resistance marker and a T7 promoter. Plasmids were transformed into E. coli BL21 (DL3) competent cells and plated on LB with kanamycin at 50mg/L. Transformants were inoculated into 50mL of autoinduction expression media (for 1L: 12g tryptone, 24g yeast extract, 20 mL 503M, 20 mL 50×5052, 2 mL 1M MgSO4, 200 mL Studier Trace metals, 100 mg kanamycin, q.s. to 1L with filtered water) in a 250mL flask. Expression cultures were grown for 20 hours at 37C with 200rpm shaking. Cells were pelleted by centrifugation at 4000×g and resuspended in a lysis buffer consisting of 25mM Tris pH 8, 300mM NaCl, and 20mM imidazole with added protease inhibitor and DNase. Cells were lysed by sonication at 85% amplitude with 8 × 15 second pulses. Lysate was separated into soluble and insoluble fractions by centrifugation at 18,000×g. Immobilized metal affinity chromatography (IMAC) was used to purify designed protein. Nickel-nitrilotriacetic acid (Ni-NTA) resin was initially equilibrated with a 5 column volumes (CV) lysis buffer. Supernatant was poured over the columns, followed by 20 CV wash buffer (25mM Tris pH 8, 400mM NaCl, 30mM imidazole). Protein was eluted using a 5CV elution buffer (25mM Tris pH 8, 300mM NaCl, 500mM imidazole). Eluate was purified by size exclusion chromatography (SEC) on an AKTA PURE FPLC system, using either a Superose 6 Increase 10/300 GL column or a Superdex 200 Increase 10/300 GL column, with Tris-buffered saline (TBS; 25mM Tris pH 8, 150mM NaCl) at a speed of 0.75mL/min. Fractions corresponding to the peak trace were collected and combined for further analysis.

### Screening novokines in Myogenic transdifferentiation assay

In a 96 well plate, 10^3 per well HFF-tet-on inducible MyoD cells in DMEM with 10% FBS were seeded. Post 2 days of seeding, for the next 4 days cells were treated with 2 ug/ml doxycycline in SMM media. The following 4 days cells are incubated with 100nM of each minibinder or their combinations. Cells were fixed in 4% paraformaldehyde [EMS #15710, diluted in phosphate buffered saline (PBS)] for 15 min, washed with PBS, and stained for Desmin/MHC/DAPI, followed by imaging at Leica spinning disk microscope, stitched (4×4) images of each 96 well at 10X was captured. In Fiji ImageJ, the desmin intensity area was thresholded and the intensity was measured as the desmin positive area.

### Length Analysis in Myogenic Transdifferentiation Assay

Using Desmin/MHC/DAPI stained 96 well plates imaged at Leica spinning disk microscope, stitched (4×4) images of each 96 well at 10X. In Fiji ImageJ, length of a desmin positive cell was determined utilizing the Freehand Line tool with a line being drawn and measured following cell curvature from tip to tip of the Desmin positive cell. Lengths were obtained for all individually distinguishable desmin positive cells in image (~100 per image). For each image the percent of measured desmin positive cells above 300 um was obtained.

### Sci-RNA-seq

Single-cell combinatorial-indexing RNA-sequencing (sci-RNA-seq) protocol is described previously (Ca0 et al., 2019; Martin et al., 2023). sci-RNA-seq relies on the following steps, thawed nuclei were permeabilized with 0.2% TritonX-100 (Sigma, #T9284) (in nuclei wash buffer) for 3 min on ice, and briefly sonicated to reduce nuclei clumping; (ii) nuclei distributed across 96-well plates. A first molecular index is introduced to the mRNA of cells within each well, with in situ reverse transcription (RT) incorporating the unique molecular identifiers (UMIs). All cells were pooled and redistributed to multiple 96-well plates in limiting numbers (e.g., 10 to 100 per well) and a second molecular index was introduced by hairpin ligation. Second strand synthesis, tagmentation, purification and indexed PCR; Library purification and sequencing is performed. All libraries were sequenced on one NovaSeq platform (Illumina). Base calls, downstream sequence processing and single-cell digital-expression matrix generation steps were similar as described in sci-RNA-seq3 paper.24 STAR79 v.2.5.2b54 aligner used with default settings and gene annotations (GRCh38-primary-assembly, gencode.v27). Uniquely mapping reads were extracted, and duplicates were removed using the UMI sequence, reverse transcription index, hairpin ligation adaptor index and read 2 end-coordinate (that is, reads with identical UMI, reverse transcription index, ligation adaptor index and tagmentation site were considered duplicates).

Sci-RNA-seq- Data Analysis All low-quality reads were removed from the data by setting UMI cutoff to greater than 200 and removing all mitochondrial reads (QC table: Figure S1F). Following Monocle3 workflow,24,33,78 data underwent normalization by size factor, preprocessing, dimension reduction (UMAP algorithm82), and unsupervised graph-based clustering analysis (Leiden Algorithm83,84). Certain clusters from the initial analysis were selected for further sub-clustering, and the previous analysis repeated. Pseudotime analysis also was done with Monocle3 following the default workflow, which included learning the graph, ordering the cells, and plotting the trajectory over UMAP. PanglaoDB,34 a curated single-cell gene expression database was utilized to explore the consensus of cell type markers used across publicly available single-cell datasets.

### Western Blotting

Lysates were boiled at 95°C for 7 minutes. After boiling, samples were vortexed for 5 seconds and spun down. For 15 well gels, 10 uL of lysate per sample was loaded per well and for 10 well gels 15 uL of sample was loaded. Gels were run for 40 minutes at 200V in a running buffer (1.44% Glycine, 0.3% Trizma, 0.1% SDS, 98.16% deionized water) using a Bio-Rad PowerPac Basic electrophoresis power supply. Following electrophoresis, gels were soaked in a transfer buffer (20% methanol, 0.24% Tris-HCl, 0.8% NaCl, 0.1% Tween-20, 78.86% deionized water) and transferred onto a nitrocellulose membrane for 12 minutes at 25V using a Bio-Rad TransBlot Turbo system. Following transfer, the membrane was blocked in 5% BSA [company and catalog number] in 1X TBST (insert components) for 1 hour at RT on a shaker, then incubated in primary antibodies overnight at 4°C on a rocker. Three 5-minute washes with 1X TBST were performed before adding HRP-conjugated secondary antibodies in 5% BSA in 1X TBST for 1 hour at RT. Three more 5-minute washes in 1X TBST were then performed. A 50/50 solution of hydrogen peroxide and HRP substrate was added to membranes immediately before imaging on a Bio-Rad ChemiDoc Touch Imaging system.

Anti-HA-Tag (Cell Signaling #3724), anti-b-actin (Cell Signaling #4970), Anti-Histone H3 (abcam #ab1791), anti-S6 (Cell Signaling #2217), anti-MHC (DSHB #MF 20), Anti-a-Actinin (abcam #ab50599)

### Immunostaining

Cells were seeded onto square glass coverslips or glass bottom 96 well imaging plates. When ready cells were washed three times for five minutes with 1X PBS, then fixed in a 4% paraformaldehyde solution for 15 minutes. Following fixation, cells were washed three times for five minutes with 1X PBS. Cells were then permeabilized in PBST (1X PBS + 0.05% Triton X) for 1 minute. After permeabilization coverslips were blocked in 1% BSA (1% BSA + 1X PBST) for 1 hour at room temperature. The appropriate primary antibody diluted in 1% BSA was then added to the cells and left overnight at 4C. The primary antibody solution was then removed and cells were given three 5-minute washes with PBST. Fluorescent secondary antibodies diluted in 1% BSA were then added to cells for 45 minutes at RT. Cells were given three 5-minute washes in PBST, then a solution of DAPI diluted in 1% BSA (1:50) was added for 15 minutes at RT. Three final 5-minute washes with PBST were given. Glycerol+n-propyl gallate mounting reagent was added directly into wells for 96 well plates, and added onto a glass slide for mounting coverslips. Stained cells were kept at 4°C until imaging was performed.

Primary antibodies- anti-Desmin (abcam #ab15200, 1:500), anti-MHC (DSHB #MF 20, 1:200), anti-ATP-B synthanse (abcam #ab14730, 1:500),

### Mito-Orange assay

Mito-Orange ( catalog no- ) was diluted to 200 nM in the appropriate prewarmed (37°C) growth media. Cells were rinsed once with prewarmed growth media then the Mito-Orange solution was added and cells were incubated at 37°C and 5% CO2 for 30 minutes. Following treatment cells

### Seahorse assay

HFF-MyoD cells were seeded into a 0.1% gelatin coated 96-well Seahorse XFe96 culture plate at a density of 20,000 cells per well. Cells were grown in HFF maintenance media until they reached 80% confluency, at which point the transdifferentiation was started. 200 uL of SMM+FBS+Dox media was added to cells at 0 and 48 hours during a 96 hour treatment period. After 96 hours media was switched to SMM-FBS media + treatments, and 200 uL was added to cells at 0 and 48 hours during a 96 hour treatment period. 2 hours prior to the seahorse assay, cells were rinsed and given 200 uL of fresh SMM-FBS media. Cells were then rinsed twice with prewarmed assay media (XF DMEM [Agilent #103575–100], L-Glutamine [Gibco #25030081] 2mM, Glucose [Sigma Aldrich G8270] 10mM, Sodium Pyruvate [11360070] 2mM) and incubated for 30 minutes in 200 uL assay media in a CO2 free incubator. After incubation cells were washed one final time, and 180 uL of assay media was added. An XFe96 Extracellular Flux Assay Kit was loaded with three treatments, 20 uL of oligomycin A [Sigma-Aldrich #75351] in port A, 22 uL of FCCP [Sigma-Aldrich #C2920] in port B, and 25 uL of Antimycin A [Sigma-Aldrich #A8674] + Rotenone [Sigma-Aldrich #R8875] in port C. Final well concentrations of the drugs was 2.5 uM Oligomycin, 1 uM FCCP, and 2.5 uM each of Rotenone + Antimycin A. Four measurements for each treatment were taken with 3 minutes of mixing, zero seconds of waiting, and 3 minutes of measuring. The assay was run at 37°C in an Agilent Seahorse XFe96 Analyzer. After the assay, the media was removed from the cell culture plate and it was frozen overnight at −20°C. The next day cells were thawed for 20 minutes, 40 uL of 0.01% SDS was added to each well and the plate was frozen at −80C for 30 minutes. Cells were then thawed for 15 minutes and 40 uL of Hoescht solution (6 ml 1M NaCl, 60 ul 100 mM EDTA, 60 ul 1M Tris HCl, 2.4ul Hoescht solution [Company, catalog no.]) was added to each each well, and the plate was left to nutate for 1 hour at 37C. A Wallac EnVision 2104 Multilabel Reader analyzer was used to take three separate measurements of the Hoescht intensity in each well. These values were then averaged and the empty corner well values subtracted, then used for normalizing the measured oxygen consumption rate values for each well. The data was analyzed using Agilent Seahorse Analytics software.

### 3-D Force experiments

#### Differentiation, purification, and expansion of iPSC-derived skeletal muscle myoblasts

iPSC-derived myoblasts (iPSC-MBs) were generated from the UC3–4 DMD null and isogenic control iPSC lines,using a multi-stage protocol adapted from previously published protocol (Smith et al., 2022). Initial expansion of induced pluripotent stem cells (iPSCs) was performed in mTeSR Plus (Stem Cell Tech) on 6-well plates (seeded at 15,000 cells/cm^2^) until cultures achieved ~40% confluence. Differentiation was initiated (Day 0) by exchanging the media for a basal formulation of DMEM/F12 (Gibco #10565018) supplemented with 1x non-essential amino acids (Gibco #11140050) and 1x Insulin-Transferrin-Selenium (ITS; Gibco #41400045). This induction medium also contained the small molecules CHIR99021 (3 μM; Axon #1386) and LDN193189 (0.2 μg/mL; Miltenyi Biotec #130–106-540). On Day 2, the culture was supplemented with 20 ng/mL bFGF (R&D Systems #3718-FB). A second media switch occurred on Day 5, removing CHIR99021 and ITS, replacing with 15% KSR (Gibco #10828028), 2 ng/mL IGF-1 (R&D Systems #291-G1), and 10 ng/mL HGF (R&D Systems #294-HGN). On Day 7, bFGF and LDN193189 were removed from the media. From Day 0 to Day 7, cultures were fed daily with 2 mL of fresh medium. From Day 7 onwards, following the removal of bFGF and LDN, media was refreshed every 48 hours.. To expand the myogenic population, Day 28 cultures were mechanically dissociated into aggregates and passaged onto Matrigel-coated T225 flasks. Expansion was carried out in Skeletal Muscle Growth Media (SKGM: high-glucose DMEM with Glutamax [Gibco #10566024], 10% FBS [Hycone #SH30071.03], 100 nM Insulin [Lonza #BE02–033E20], 40 ng/mL FGF-2 [R&D Systems #3718-FB], and 1 μM Dexamethasone [Sigma #D4902]), with media changes every 2–3 days. Finally, on Day 32, single-cell suspensions were generated by filtration (40 μm) and sorted via fluorescence-activated cell sorting (FACS) to isolate Nerve Growth Factor Receptor-positive (NGFR+) progenitors (Biolegend #345108). Experiments utilized cells from passages 4–5.

#### Generation of 3D engineered muscle tissues (EMTs)

Engineered muscle tissues (EMTs) were generated using the Mantarray platform (Curi Bio) following an adapted fabrication protocol. A co-culture cell pellet was prepared by combining iPSC-derived myoblasts and human dermal fibroblasts (Lonza, CC-2511) at a 9:1 ratio. Cells were resuspended to achieve a final density of 420,000 cells per 60 μL hydrogel mixture composed of 1.3 mg/mL rat tail collagen I (Sigma-Aldrich, 08–115), 20% Matrigel (Corning, 356231), 10% MEM (Gibco #11095080), and 10% high-glucose DMEM with Glutamax. This cell-hydrogel suspension was pipetted directly into the Mantarray casting trenches and polymerized for 60 minutes at 37 °C (5% CO_2_). Following polymerization, 1 mL of SKGM was added per well for 24-hours. Constructs were then transferred to fresh 24-well plates containing 1.5 mL of differentiation medium (high-glucose DMEM with Glutamax and 2% Horse Serum [Gibco #16050122]). For treatment groups, this medium was supplemented with 100 nM C6 cocktail. The time of transfer to differentiation medium was designated as differentiation Day 0. Maintenance involved replacing half the media volume (± C6 cocktail) every 2–3 days.

#### Measurement of EMT contraction

Contractile function was assessed using the Mantarray platform (Curi Bio). The 24-well EMT plate was loaded into the instrument’s measurement chamber, maintained at 37°C, and a stimulation lid assembly was inserted to position electrodes within each well, enabling simultaneous electrical pacing of all tissues. Magnetic field flux was recorded at a sampling rate of 100 Hz and converted to post deflection (mm) using the manufacturer’s proprietary magnet localization algorithm. These deflection data were subsequently converted to contractile force to generate force-time traces for analysis. Tissues underwent three distinct stimulation protocols utilizing biphasic electrical pulses (5 ms duration, 100 mA). All raw force data were processed using the Engineered Muscle Contractility Analysis Pipeline (EMCAP) version 1.0, a custom open-source Python based peak-finding and analysis package (Barrett, 2026). Twitch force and kinetics were evaluated using a Twitch Protocol (0.2 Hz), consisting of 10 individual pulses separated by 5-second rest intervals; active twitch force was calculated as the average of these ten contractions. Tetanic force and kinetics were determined using a Force-Frequency Protocol, where tissues were subjected to 2-second stimulation trains at increasing frequencies (1, 2, 3, 5, 10, 15, 20, 30, 40, 60, 80, and 100 Hz), with a 16-second rest period between each step.

#### EMT Immunohistochemistry and immunocytochemistry

To determine the effective physiological cross-sectional area (CSA) for specific force calculations and to assess myotube morphology, EMTs were fixed on Day 14 in 4% paraformaldehyde (24 h, 4°C). Tissues were cryoprotected in 20% sucrose (w/w) for 24 hours, embedded in OCT compound, and stored at −80°C. Transverse cryosections (30 μm) were blocked for 1 hour at room temperature in blocking buffer (2% BSA, 5% goat serum, 0.25% Triton X-100). Sections were incubated overnight at 4°C with primary antibodies against Myosin Heavy Chain (MF-20, 1 μg/mL) and Dystrophin (Abcam ab15277, 2 μg/mL). Following primary incubation, sections were stained Goat anti-mouse 488, Goat anti-rabbit 546, Phalloidin 647, and Hoechst (overnight, 4°C) and imaged using a Yokogawa W1 spinning disk confocal microscope equipped with a 20x air objective.

Image processing was automated using custom ImageJ/Fiji macros. Image files were imported and aligned using the SIFT (Scale-Invariant Feature Transform) algorithm to correct for channel drift. To accurately segment individual myotubes, a “fusion” image was generated by averaging the background-subtracted MF-20 and Phalloidin signals. This fusion strategy was critical for maintaining segmentation fidelity. These fused images were segmented using StarDist plugin, a deep-learning-based object detection tool (https://arxiv.org/abs/2203.02284). Individual and sum of myotube CSA was calculated from these segmented regions and used to normalize active force to specific force (mN/mm^2^).

#### Statistical analysis-

Two-sided Student’s t-test was performed to determine p-value. For phosphoproteomics analyses, Welch’s t-tests were used to assess statistical significance followed by Benjamini-Hochberg procedure to correct for multiple hypothesis testing.

## Supplementary Material

Supplement 1
