# Supplementary material for: Designed Minibinders Rewire Receptor Signaling to Enable Functional Human Myogenic Reprogramming
Source: bioRxiv. 2026 Apr 27:2026.04.26.720818. Preprint. [Version 1] doi: 10.64898/2026.04.26.720818 (PMC13142334; doi:10.64898/2026.04.26.720818)
Supplement: Supplement 1 [file media-1.pdf]

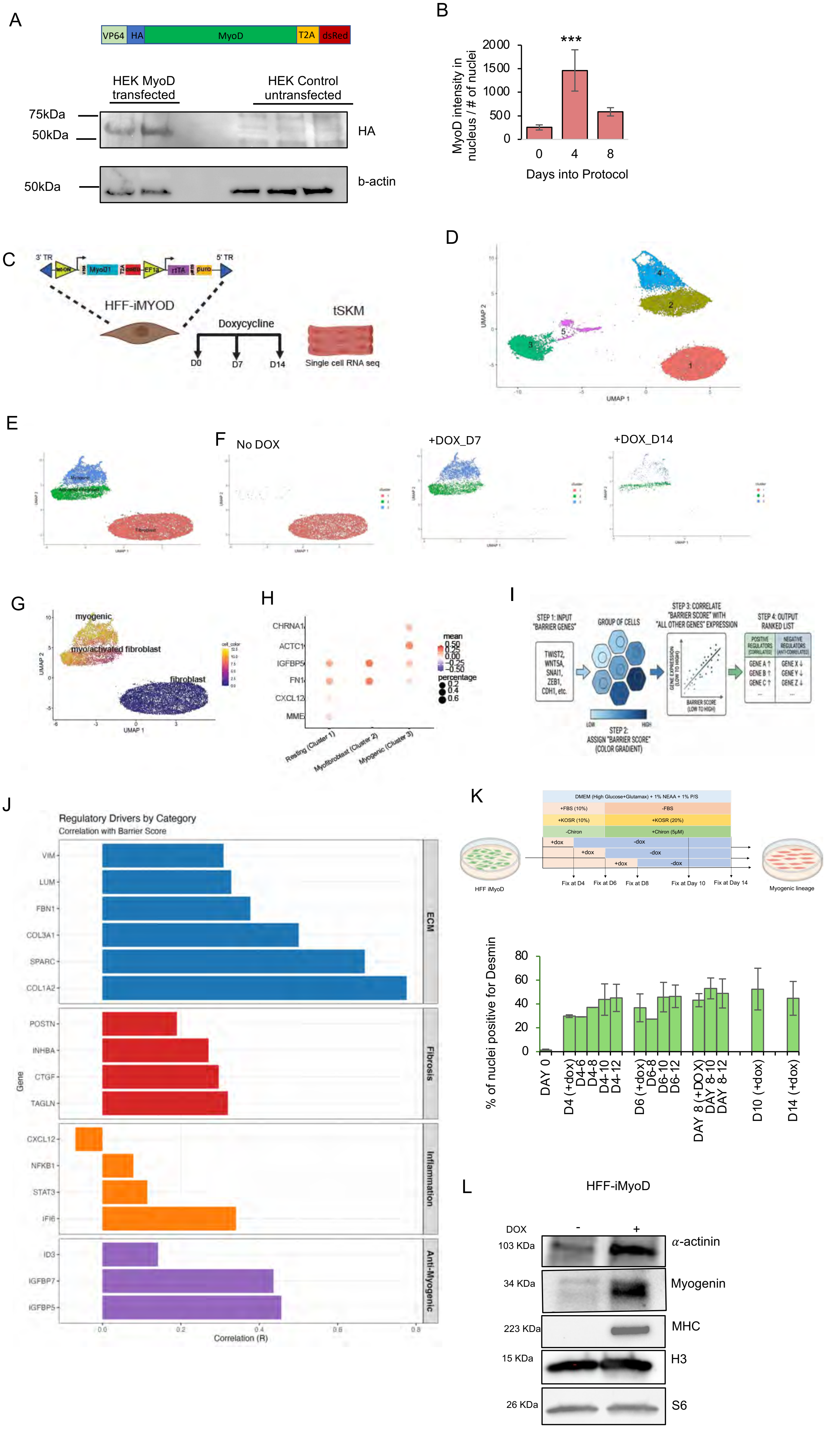

A
VP64
HA
MyoD
T2A
dsRed
HEK MyoD transfected
HEK Control untransfected
75kDa
50kDa
HA
50kDa
b-actin
B
MyoD intensity in nucleus / # of nuclei
***
Days into Protocol
C
HFF-iMYOD
Doxycycline
D0
D7
D14
tSKM
Single cell RNA seq
D
UMAP 1
UMAP 2
E
F
No DOX
+DOX_D7
+DOX_D14
G
myogenic
myo/activated fibroblast
fibroblast
UMAP 1
UMAP 2
H
CHRNA1
ACTC1
IGFBP5
FN1
CXCL12
MME
mean
percentage
Resting (Cluster 1)
Myofibroblast (Cluster 2)
Myogenic (Cluster 3)
I
STEP 1: INPUT "BARRIER GENES"
TWIST2, WNT5A, SNAI1, ZEB1, CDH1, etc.
GROUP OF CELLS
STEP 2: ASSIGN "BARRIER SCORE" (COLOR GRADIENT)
STEP 3: CORRELATE "BARRIER SCORE" WITH "ALL OTHER GENES" EXPRESSION
STEP 4: OUTPUT RANKED LIST
J
Regulatory Drivers by Category
Correlation with Barrier Score
VIM
LUM
FBN1
COL3A1
SPARC
COL1A2
ECM
POSTN
INHBA
CTGF
TAGLN
Fibrosis
CXCL12
NFKB1
STAT3
IFI6
Inflammation
ID3
IGFBP7
IGFBP5
Anti-Myogenic
Gene
Correlation (R)
K
DMEM (High Glucose+Glutamax) + 1% NEAA + 1% P/S
+FBS (10%)
-FBS
+KOSR (10%)
+KOSR (20%)
-Chiron
+Chiron (5µM)
+dox
-dox
HFF iMyoD
Myogenic lineage
Fix at D4
Fix at D6
Fix at D8
Fix at Day 10
Fix at Day 14
% of nuclei positive for Desmin
DAY 0
D4 (+dox)
D4-6
D4-8
D4-10
D4-12
D6 (+dox)
D6-8
D6-10
D6-12
DAY 8 (+DOX)
DAY 8-10
DAY 8-12
D10 (+dox)
D14 (+dox)
L
HFF-iMyoD
DOX
103 KDa
α-actinin
34 KDa
Myogenin
223 KDa
MHC
15 KDa
H3
26 KDa
S6

Supplementary figures 1

(A) A schematic showing the recombinant MyoD transcription factor used in our study with N -ter VP64 transactivation domain, HA tag and on c-term linked to dsRED with a cleavable T2A site. Western blot analysis shows that HEK cells transfected with MyoD plasmid treated with doxycycline show expression of MyoD protein, Beta actin is used as a housekeeping gene. (B) A bar graph showing the Myod/no of nuclei intensity in HFF-iMYOD cells immunostained for MyoD antibody and DAPI.
(C) tet-on inducible MyoD Human Foreskin FIbroblast were treated with Doxycycline for 7 days and 14 days, to reprogram into muscle cells, where single cell nuclei sequencing was performed. (D) UMAP showing single nuclei RNA sequencing in Fig.1A. in all treatments.
(E) UMAP showing single nuclei RNA sequencing of all treatments excluding the unwanted cell types. (F) UMAP showing D0 (resting fibroblast), D7 (tKM) and d14 (tSKM) cells.
(G)Pseudotime analysis using Monocle3. (H) A dot plot showing resting, activated fibroblast, and muscle markers in the three specific clusters. (I) A schematic showing the antimyogenic or barriers of myogenic gene expression was scored to calculate the barrier score of myofibroblast/ activated fibroblast versus the myogenic cluster. (J) Regulatory drivers of anti-myogenic (barrier score) of myofibroblast/ activated fibroblast versus the myogenic cluster.
(K) A schematic showing different time points [d4,d6,d8,d10,d14] of Doxycyline (dox) treatment followed by release in the media lacking serum and dox treatment [d0,d6,d8,d10,d12]. A bar graph showing the various time point treatment and conversion efficiency (% of Desmin positive nuclei) was calculated from cells fixed and immunostained for the Desmin and Myosin heavy chain and DAPI.
(L) Western blot analysis of day 0 and day 8 tSKM showing muscle marker protein expression level of alpha-actinin, Myosin heavy chain(MHC) and Myogenin and two housekeeping genes Histone3 and S6.(J) cluster 2 versus cluster 3 comparison of barriers for the transition to muscle.

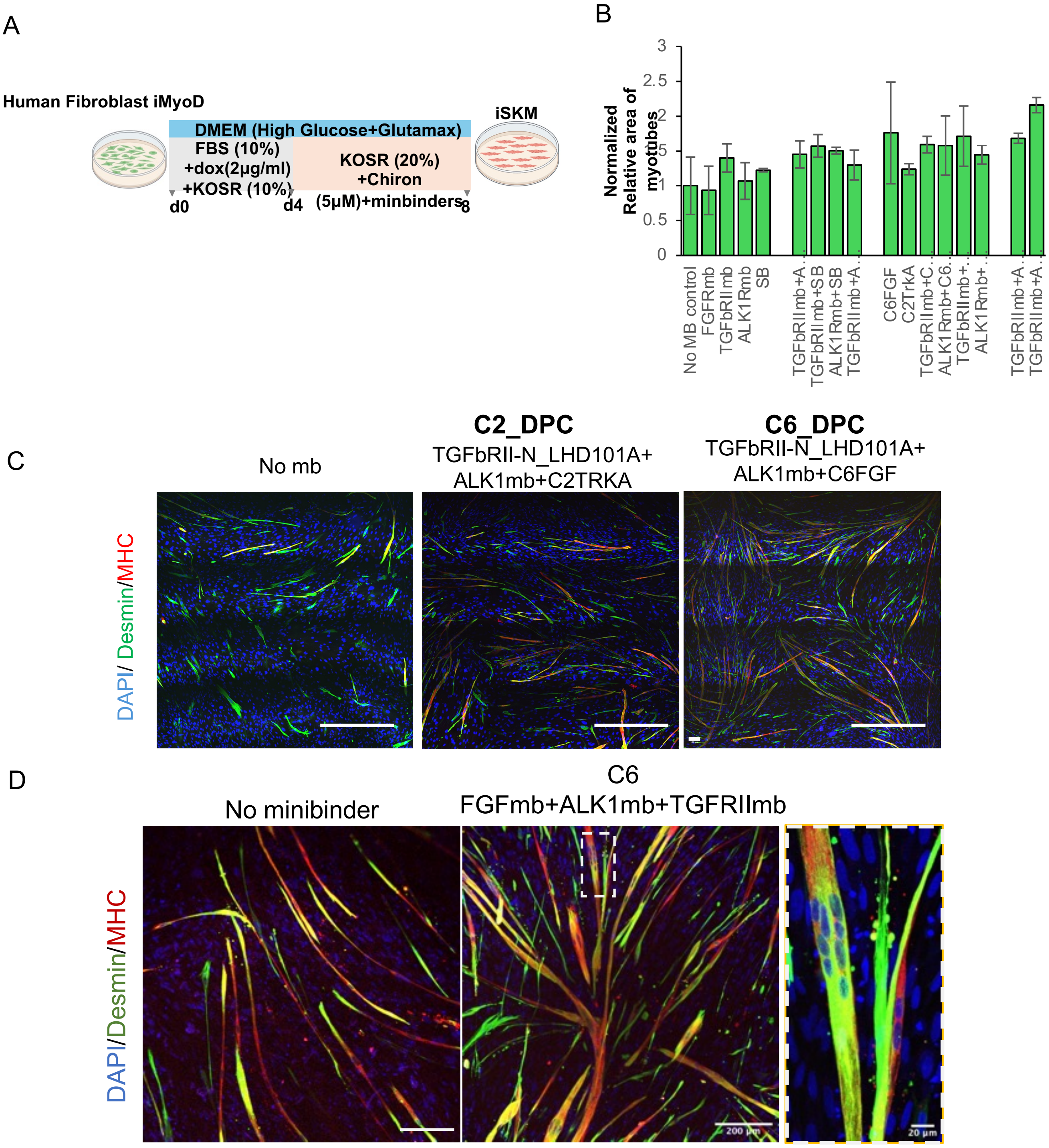

Supplementary figures 2

1.A schematic showing fibroblast to tSKM conversion in presence of design protein cocktail treatment.
2. A bar graph showing myotube area calculated using imageJ from cells stained for Desmin and DAPI where the Desmin area over the same no of nuclei was calculated under different design minibinders treatments in d8 tSKM.
3.Immunofluorescence images of under no mb (control) and C2-DPC and C6-DPC treated d8 tSKM immunostained for the Desmin (green), MHC(red) and DAPI (DAPI) in 96 well where 4X by 4X stitched images are shown.
Immunofluorescence images of under no mb (control) and C6-DPC treated d8 tSKM immunostained for the Desmin (green), MHC(red) and DAPI (DAPI) showing multinucleated myotubes. Scale bar=200um and 20um

Figure-S3

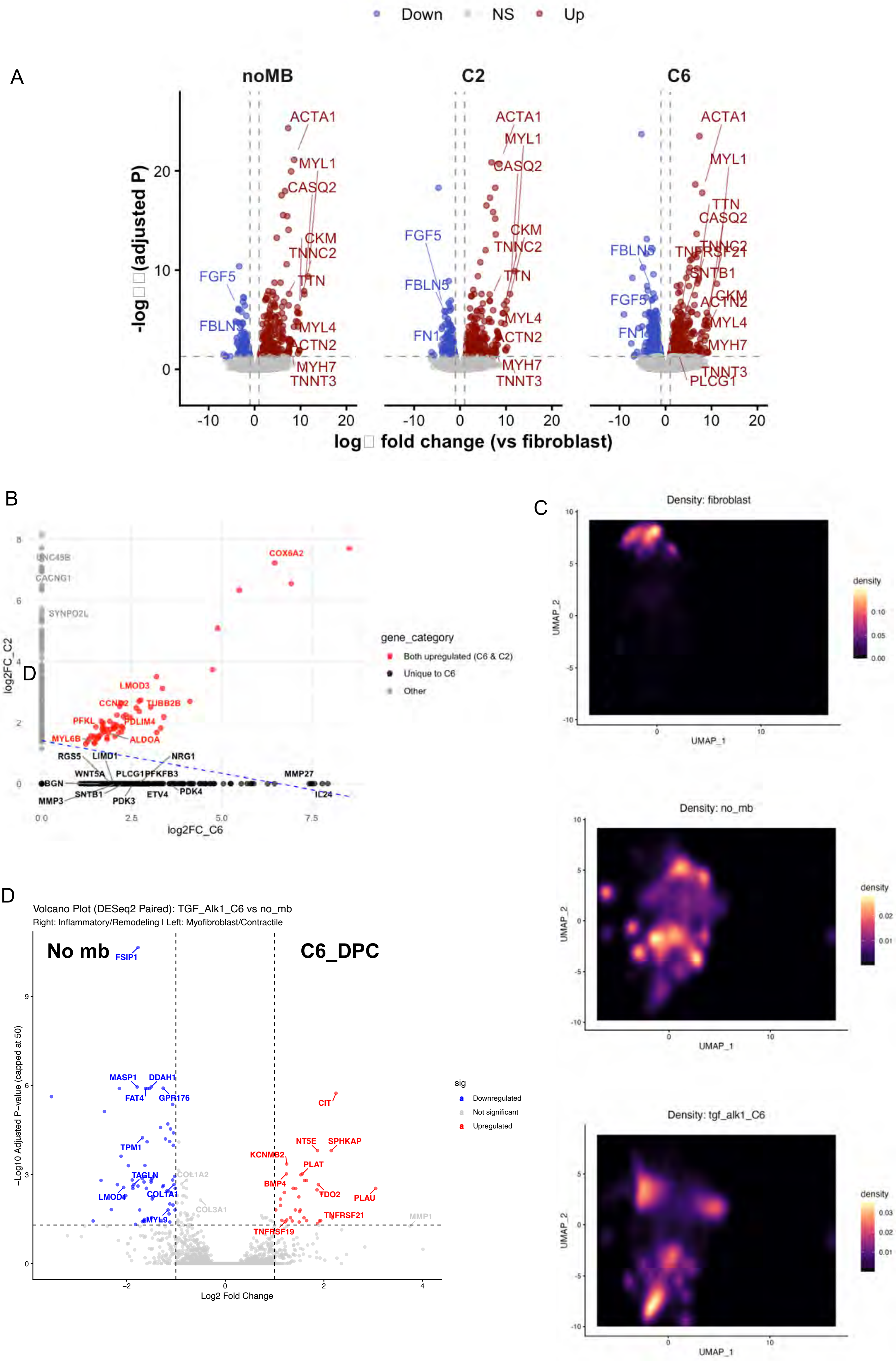
Down
NS
Up
A
noMB
C2
C6
-log□□(adjusted P)
log□ fold change (vs fibroblast)
ACTA1
MYL1
CASQ2
CKM
TNNC2
TTN
MYL4
ACTN2
MYH7
TNNT3
FGF5
FBLN5
FN1
TNFRSF21
SNTB1
PLCG1
B
gene_category
Both upregulated (C6 & C2)
Unique to C6
Other
log2FC_C2
log2FC_C6
COX6A2
UNC45B
CACNG1
SYNPO2L
LMOD3
CCNB2
TUBB2B
PFKL
PDLIM4
MYL6B
ALDOA
RGS5
LIMD1
NRG1
WNT5A
PLCG1
PFKFB3
MMP27
BGN
SNTB1
ETV4
PDK4
IL24
MMP3
PDK3
C
Density: fibroblast
Density: no_mb
Density: tgf_alk1_C6
density
UMAP_1
UMAP_2
D
Volcano Plot (DESeq2 Paired): TGF_Alk1_C6 vs no_mb
Right: Inflammatory/Remodeling | Left: Myofibroblast/Contractile
No mb
C6_DPC
FSIP1
MASP1
DDAH1
FAT4
GPR176
TPM1
TAGLN
COL1A2
LMOD1
COL1A1
COL3A1
MYL9
CIT
NT5E
SPHKAP
KCNMB2
PLAT
BMP4
TDO2
PLAU
TNFRSF21
TNFRSF19
MMP1
−Log10 Adjusted P−value (capped at 50)
Log2 Fold Change
sig
Downregulated
Not significant
Upregulated

Supplementary figures3

(A)Volcano map showing skeletal muscle gene expression upregulated in no mb, C2-DPC and C6-DPC 8day tSKM over the fibroblast. (B) Unique and common (red) gene expression found in C2-DPC and C6-DPC 8day tSKM over the fibroblast. (C) Density of nuclei in single nuclei sequencing of fibroblast, no mb and C6-DPC treated mononucleated cells. (D) A volcano map showing pseudobulk data where upregulated and downregulated genes are represented in C6-DPC versus no mb mononucleated cells upon 8 days of transdifferentiation

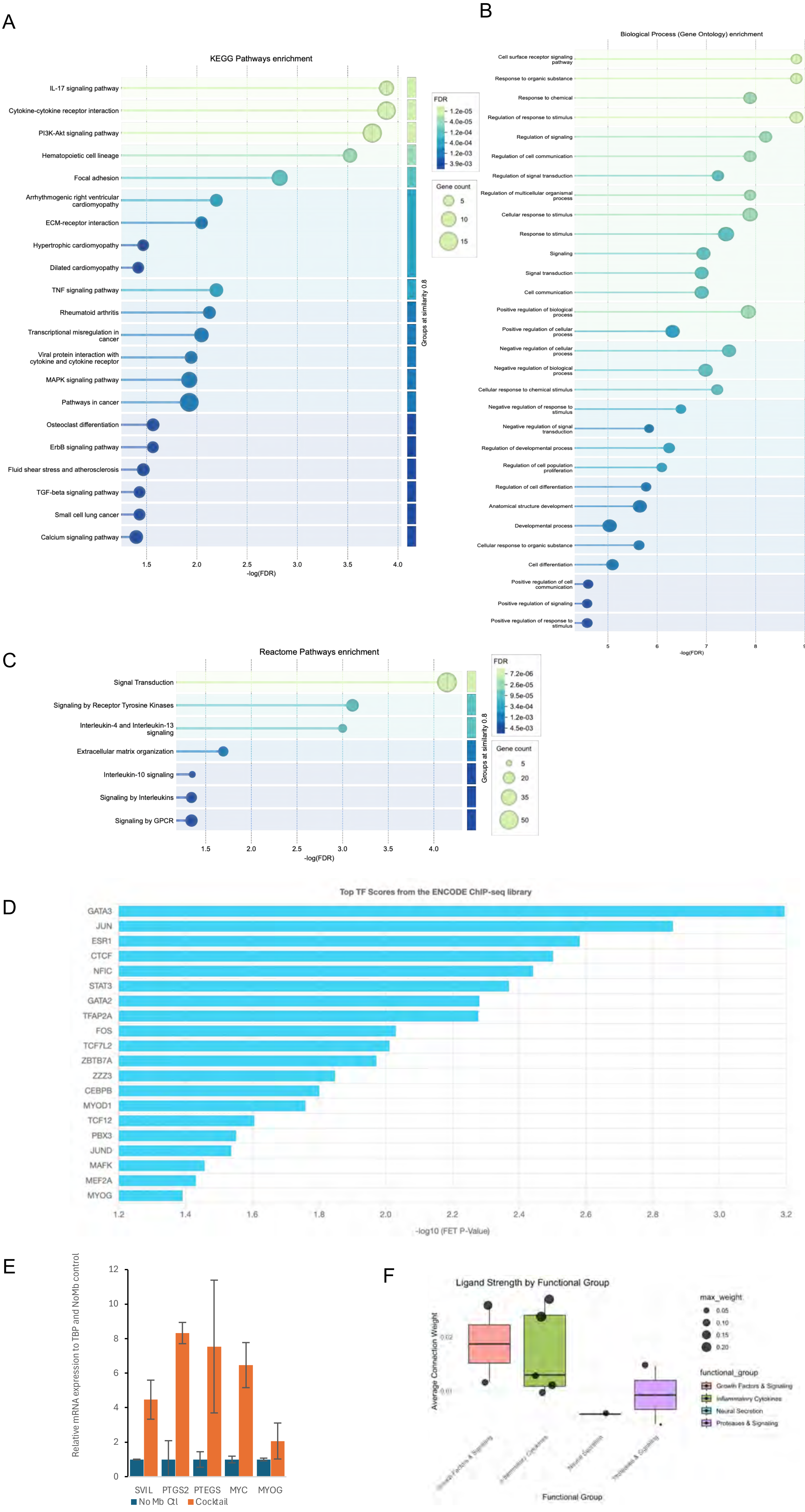
A
KEGG Pathways enrichment
IL-17 signaling pathway
Cytokine-cytokine receptor interaction
PI3K-Akt signaling pathway
Hematopoietic cell lineage
Focal adhesion
Arrhythmogenic right ventricular cardiomyopathy
ECM-receptor interaction
Hypertrophic cardiomyopathy
Dilated cardiomyopathy
TNF signaling pathway
Rheumatoid arthritis
Transcriptional misregulation in cancer
Viral protein interaction with cytokine and cytokine receptor
MAPK signaling pathway
Pathways in cancer
Osteoclast differentiation
ErbB signaling pathway
Fluid shear stress and atherosclerosis
TGF-beta signaling pathway
Small cell lung cancer
Calcium signaling pathway
1.5
2.0
2.5
3.0
3.5
4.0
-log(FDR)
FDR
1.2e-05
4.0e-05
1.2e-04
4.0e-04
1.2e-03
3.9e-03
Gene count
5
10
15
Groups at similarity 0.8
B
Biological Process (Gene Ontology) enrichment
Cell surface receptor signaling pathway
Response to organic substance
Response to chemical
Regulation of response to stimulus
Regulation of signaling
Regulation of cell communication
Regulation of signal transduction
Regulation of multicellular organismal process
Cellular response to stimulus
Response to stimulus
Signaling
Signal transduction
Cell communication
Positive regulation of biological process
Positive regulation of cellular process
Negative regulation of cellular process
Negative regulation of biological process
Cellular response to chemical stimulus
Negative regulation of response to stimulus
Negative regulation of signal transduction
Regulation of developmental process
Regulation of cell population proliferation
Regulation of cell differentiation
Anatomical structure development
Developmental process
Cellular response to organic substance
Cell differentiation
Positive regulation of cell communication
Positive regulation of signaling
Positive regulation of response to stimulus
5
6
7
8
9
-log(FDR)
FDR
1.0e-09
1.0e-08
7.0e-08
5.0e-07
3.0e-06
2.0e-05
Gene count
40
80
120
Groups at similarity 0.8
C
Reactome Pathways enrichment
Signal Transduction
Signaling by Receptor Tyrosine Kinases
Interleukin-4 and Interleukin-13 signaling
Extracellular matrix organization
Interleukin-10 signaling
Signaling by Interleukins
Signaling by GPCR
1.5
2.0
2.5
3.0
3.5
4.0
-log(FDR)
FDR
7.2e-06
2.6e-05
9.5e-05
3.4e-04
1.2e-03
4.5e-03
Gene count
5
20
35
50
Groups at similarity 0.8
D
Top TF Scores from the ENCODE ChIP-seq library
GATA3
JUN
ESR1
CTCF
NFIC
STAT3
GATA2
TFAP2A
FOS
TCF7L2
ZBTB7A
ZZZ3
CEBPB
MYOD1
TCF12
PBX3
JUND
MAFK
MEF2A
MYOG
1.2
1.4
1.6
1.8
2.0
2.2
2.4
2.6
2.8
3.0
3.2
-log10 (FET P-Value)
E
Relative mRNA expression to TBP and NoMb control
12
10
8
6
4
2
0
SVIL
PTGS2
PTEGS
MYC
MYOG
No Mb Ctl
Cocktail
F
Ligand Strength by Functional Group
Average Connection Weight
0.02
0.01
Functional Group
max_weight
0.05
0.10
0.15
0.20
functional_group
Growth Factors & Signaling
Inflammatory Cytokines
Neural Secretion
Proteases & Signaling

Supplementary figures 4

(A) KEGG analysis of genes upregulated in C6-DPC versus no minibinder.
(B) Biological Process GO terms, analysis of genes upregulated in C6-DPC versus no minibinder. (C) Reactome analysis of genes upregulated in C6-DPC versus no minibinder. (D) Top transcription factors based on their score from ENCODE ChIP-seq library in upregulated gene expression in C6-DPC (E) qPCR mRNA expression of genes in C6-DPC over no mb tSKM (F) functional grouping of upregulated genes in C6-DPC over no mb bulk sequencing.
